# A comprehensive analysis of tumor-stromal collagen in relation to pathological, molecular, and immune characteristics and patient survival in pancreatic ductal adenocarcinoma

**DOI:** 10.1007/s00535-023-02020-8

**Published:** 2023-07-21

**Authors:** Shigeto Ashina, Atsuhiro Masuda, Kohei Yamakawa, Tsuyoshi Hamada, Masahiro Tsujimae, Takeshi Tanaka, Hirochika Toyama, Keitaro Sofue, Hideyuki Shiomi, Arata Sakai, Takashi Kobayashi, Shohei Abe, Masanori Gonda, Shigeto Masuda, Noriko Inomata, Hisahiro Uemura, Shinya Kohashi, Kae Nagao, Yoshiyuki Harada, Mika Miki, Noriko Juri, Yosuke Irie, Maki Kanzawa, Tomoo Itoh, Jun Inoue, Toshio Imai, Takumi Fukumoto, Yuzo Kodama

**Affiliations:** 1 Division of Gastroenterology, Department of Internal Medicine, Kobe University Graduate School of Medicine, 7-5-1 Kusunoki-Cho, Chuo-ku, Kobe, Hyogo 650-0017 Japan; 2 Department of Gastroenterology, Graduate School of Medicine, The University of Tokyo Hospital, 7-3-1 Hongo, Bunkyo-ku, Tokyo 113-8655 Japan; 3 Division of Hepato-Biliary-Pancreatic Surgery, Department of Surgery, Kobe University Graduate School of Medicine, 7-5-1 Kusunoki-Cho, Chuo-ku, Kobe, Hyogo 650-0017 Japan; 4 Department of Radiology, Kobe University Graduate School of Medicine, 7-5-1 Kusunoki-Cho, Chuo-ku, Kobe, Hyogo 650-0017 Japan; 5 Division of Gastroenterology and Hepatobiliary and Pancreatic Diseases, Department of Internal Medicine, Hyogo College of Medicine, 1-1 Mukogawa-Cho, Nishinomiya, Hyogo 650-0017 Japan; 6 Division of Diagnostic Pathology, Kobe University Graduate School of Medicine, 7-5-1 Kusunoki-Cho, Chuo-ku, Kobe, Hyogo 650-0017 Japan

**Keywords:** Tumor-stromal collagen, Major driver gene, Prognosis, Tumor-infiltrating lymphocytes, Pancreatic ductal adenocarcinoma

## Abstract

**Background:**

Abundant collagen deposition is a hallmark of pancreatic ductal adenocarcinomas (PDACs). This study clarified the interactive relationship between tumor-stromal collagen, molecular and immune characteristics, and tumor pr ogression in human PDAC.

**Methods:**

We performed a comprehensive examination using an integrative molecular pathological epidemiology database on 169 cases with resected PDAC . The amount of tumor-stromal collagen was quantified through digital imaging analysis for Elastica van Gieson-stained whole-section tumor slides. We analyzed the association of tumor-stromal collagen with gene alterations (*KRAS*, *TP53*, *CDKN2A*/p16, and *SMAD4*), immune parameters (CD4^+^ tumor-infiltrating lymphocytes [TILs], CD8^+^ TILs, FOXP3^+^ TILs, and tertiary lymphoid structures), and patient prognosis.

**Results:**

Low amounts of tumor-stromal collagen were associated with poor differentiation (multivariable OR = 3.82, 95%CI = 1.41–12.2, *P = *0.008) and *CDKN2A*/p16 alteration (OR [95%CI] = 2.06 [1.08–4.02], *P = *0.03). Tumors with low collagen levels had shorter overall survival (HR [95%CI] = 2.38 [1.59–3.56], *P < *0.0001). In the S-1 and gemcitabine (GEM) treatment groups, low tumor-stromal collagen was linked to poor prognosis of patients with PDAC (S-1 group: multivariable HR [95%CI] = 2.76 [1.36–5.79],* P = *0.005; GEM group: multivariate HR [95%CI] = 2.91 [1.34–6.71], *P = *0.007). Additionally, low amounts of tumor-stromal collagen were also linked to low levels of CD4^+^ TILs (*P = *0.046), CD8^+^ TILs (*P = *0.09), and tertiary lymphoid structures (*P = *0.001).

**Conclusions:**

Tumor-stromal collagen deposition may play a crucial role in modulating tumor-immune microenvironment and determining response to adjuvant chemotherapy and patient survival outcomes.

**Supplementary Information:**

The online version contains supplementary material available at 10.1007/s00535-023-02020-8.

## Introduction

Pancreatic ductal adenocarcinoma (PDAC) is a malignancy with a poor patient prognosis and a 10% 5-year survival rate [1]. Despite the development of conventional cytotoxic chemotherapy, molecular target therapies , and immunotherapy, PDAC remains largely resistant to treatment [2–4]. The tumor microenvironment (TME), characterized by dense stromal fibrosis and immunosuppressive cell populations, has been attributed to this highly treatment-resistant PDAC phenotype [5–8]. However, there exists a considerable inter-tumor heterogeneity in stromal fibrosis. A deeper understanding of the pathogenesis underlying the desmoplastic stroma could help us stratify patients based on the tumor progression risk and develop new treatment strategies.

The TME of PDAC comprises abundant extracellular matrix (ECM) proteins, mainly type I collagen [9], and various types of cells, such as immune cells, endothelial cells, and cancer-associated fibroblasts (CAFs) [10]. Of these CAFs, ACTA2 (α-smooth muscle actin [α-SMA])^+^ myofibroblasts play a pivotal role in producing ECM proteins [11, 12]. Mechanistic data attest that the dense tumor stroma in PDAC potentially enhance chemoresistance and tumor progression by causing a hypoxic TME, thereby impeding drug delivery [13–16]. However, recent studies have reported conflicting data supporting increased tumor stroma as an inhibitor of PDAC progression [17, 18]. In a PDAC mouse model, blockage of the Hedgehog pathway that activates CAFs resulted in the reduction of the tumor stroma and tumor differentiation, leading to accelerated PDAC progression [18, 19]. Deletion of type I collagen in α-SMA^+^ myofibroblasts also resulted in poorly differentiated carcinoma and a poor prognosis [11]. Therefore, whether the fibrotic TME exhibits a promoting or suppressing effect on PDAC cells remains controversial.

The fibrosis status of the TME in PDAC may determine the effectiveness to anti-tumor immunotherapy. Some studies have revealed that an increased number of CD4^+^ helper T cells, and CD8^+^ cytotoxic T cells, known as tumor-infiltrating lymphocytes (TILs) are positively correlated with a favorable prognosis in patients with PDAC [20, 21]. Previously, it was believed that fibrosis within the TME of PDAC impeded the recruitment of these TILs [22, 23]. However, a recent research suggested that dense fibrosis within the TME is associated with increased TILs and improved progression-free survival [24]. Additionally, the formation of tertiary lymphoid structures (TLSs) i.e., aggregates of immune cells in non-lymphoid tissues [25], may also regulate tumor immunity in PDAC [26] and have been reportedly linked to a favorable prognosis and response to neoadjuvant chemotherapy [27, 28]. These findings imply that fibrosis within the TME of PDAC may impact patient outcomes through tumor immunity regulation. Furthermore, alterations of four major driver genes (*KRAS*, *TP53*, *CDKN2A/*p16, and *SMAD4*) have also been associated with patient prognosis in PDAC [29, 30]. However, a comprehensive examination of the relationship between fibrosis within the TME, TILs, and tumor characteristics, including driver gene alterations, has not been conducted in human PDAC.

Therefore, we aimed to analyze an integrative database of 169 patients with resected PDAC to examine the associations of tumor-stromal collagen with pathological, molecular, and immune characteristics and patient survival.

## Methods

### Patients

In this study, we initially identified 234 patients with PDAC who underwent pancreatectomy between April 2008 and March 2017 at Kobe University Hospital. We next excluded 19 patients who had adenocarcinoma originating from intraductal papillary mucinous neoplasms (*N = *8), adenosquamous carcinoma (*N = *7), anaplastic carcinoma (*N = *4), or mucinous carcinoma (*N = *2). We further excluded patients with stage IV PDAC (*N = *15), those who underwent preoperative radiation (*N = *13), and those without available formalin-fixed paraffin-embedded (FFPE) samples (*N = *15) for the analysis. Thus, we analyzed 169 conventional patients with PDAC as the final study population.

### Data collection

We retrospectively collected the following patient information from medical records: age, sex, body mass index (BMI) at surgery, family history of PDAC, alcohol consumption (< 50 g/day or ≥ 50 g/day regularly), smoking history (presence: those who had smoked at or before the time of surgery or absence: those who had never smoked in their lifetime), diabetes mellitus (presence or absence), serum carbohydrate antigen 19–9 (CA19-9) (< 37 U/ml or ≥ 37 U/ml), serum carcinoembryonic antigen (< 5 U/ml or ≥ 5 U/ml), neoadjuvant chemotherapy (presence or absence), and adjuvant chemotherapy (presence or absence). We also collected tumor information on pathological stage (UICC TNM classification 8^th^ edition), T factor, tumor location, histological grade, and residual tumor status.

This study was performed in accordance with the Declaration of Helsinki and was approved by the Ethics Committees of the Kobe University Hospital (no.180235). The need for informed consent was waived due to the retrospective nature of this study design.

### Immunohistochemistry (IHC) and Elastica van Gieson (EVG) staining

Five-micron-thick tissue sections were processed from the FFPE samples with the largest tumor area. The IHC process was carried out using a VENTANA BenchMark GX (Roche Diagnostics, Basel, Switzerland) and primary antibodies specific for *TP53* (Santa Cruz Biotechnology, Dallas, TX, USA, catalog number: sc-47698), *CDKN2A/*p16 (Roche Diagnostics, catalog number: 705–4713), *SMAD4* (Santa Cruz Biotechnology, catalog number: sc-7966), CD4 (Leica Biosystems, Wetzlar, Germany, catalog number: CD4-368-L-CE), CD8 (Roche Diagnostics, catalog number: 790–4460), FOXP3 (Abcam, Cambridge, UK, catalog number: ab20034), and α-SMA (Santa Cruz Biotechnology, catalog number: sc-53142). EVG staining, a specific method for collagen visualization, was performed using an Elastic Stain Kit (Abcam, catalog number: ab150667) on the same section used for IHC, according to the manufacturer's protocol.

### Evaluation of the tumor-stromal collagen amount, α-SMA^+^ myofibroblast, and cancer cell components in PDAC

**Fig. 1:**
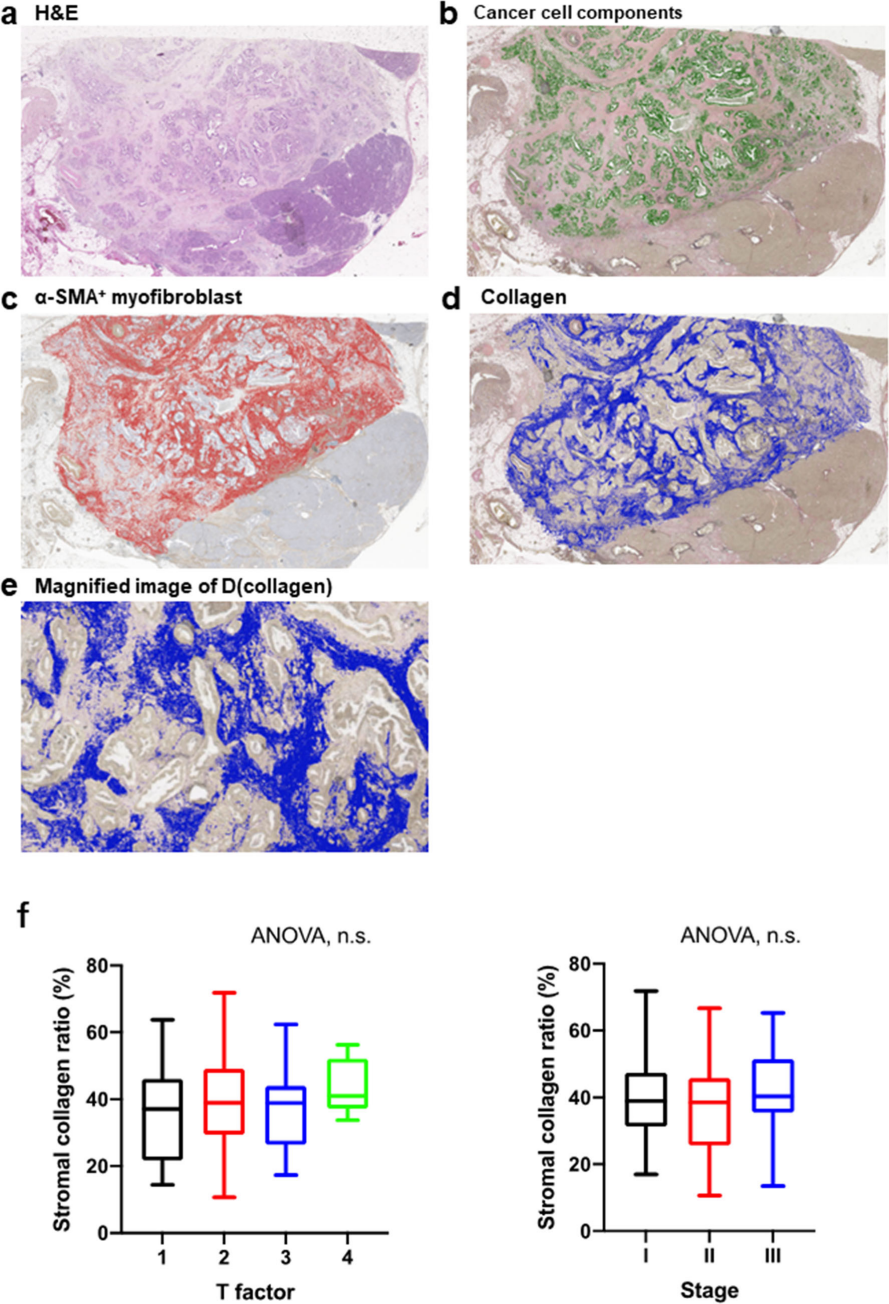
An illustration of the image analysis used to quantify cancer cell component and fibrosis status of the tumor microenvironment in pancreatic ductal adenocarcinoma (PDAC). **a** Illustrative image of a section of excised PDAC stained with hematoxylin and eosin. **B, c** The area of the cancer cell component within the tumor in an Elastica van Gieson (EVG)-stained section is depicted in green, while the area of α-smooth muscle actin positive myofibroblasts in an immunohistochemical section is in red, **d, e** The area of collagen within the tumor stroma in an EVG-stained section is illustrated in blue. **f** A correlation between the ratio of stromal collagen and T factor (left) or pathological stage (right) is illustrated

Hematoxylin and eosin (H&E) stained, EVG stained, and α-SMA stained slides were digitally scanned using a Plustek OpticFilm (Plustek, Taiwan, China) and imported into Adobe Photoshop CC2019 software (Adobe Inc., San Jose, CA) (Fig. 1a).

Each area in a tissue section was defined based on the following criteria, and the number of pixels in each area was calculated: The “total tumor area” refers to the whole section, excluding the adipose tissue, large blood vessels, and other accessory tissues (Fig. 1a). The “cancer cell component area” was extracted manually (Fig. 1b). The “tumor stroma area” was calculated by subtracting the “cancer cell component area” from the “total tumor area.” The “α-SMA^+^ area” denotes the brown intensity area in the “tumor stroma area” of an α-SMA-stained section (Fig. 1c). The “tumor-stromal collagen area” indicates the red intensity area in the “tumor stroma area” of an EVG-stained section (Fig. 1d, and e).

The proportion of each area was calculated according to the following criteria, and all the cases were classified into two groups (high and low) based on each median area proportion: The proportion of “αSMA + area” = “αSMA + area”/ “tumor stroma area.” The proportion of “tumor-stromal collagen area” = “tumor-stromal collagen area”/“tumor stroma area.” The proportion of “cancer cell component” = “cancer cell component”/“total tumor area. This method allows for a quantitative evaluation of the entire tumor, including its intra-heterogeneity, which cannot be assessed by magnified imaging analysis.

### Next-generation sequencing (NGS) analysis

In accordance with the method described previously [31], DNA was extracted from FFPE sections of PDAC. The extracted DNA was used for targeted amplicon NGS and copy number variation (CNV) detection using droplet digital PCR (ddPCR). For NGS, the obtained sequence data has been registered in the DNA Data Bank of Japan database under accession number DRA011317.

### CNV detection using ddPCR

CNV of *TP53*, *CDKN2A*, and *SMAD4* was analyzed using the QX200 Droplet Digital PCR System (Bio-Rad Laboratories, Munich, Germany) according to previously published methods [32, 33]. *TP53* (assay ID: dHsaCP1000586), *CDKN2A* (assay ID: dHsaCP1000581), *SMAD4* (assay ID: dHsaCP2500468), and *AP3B1*, as a reference (assay ID: dHsaCP2500348), were used for CNV assay using ddPCR. Samples with copy number values < 1.4 were defined as a “deletion.”

### Final classification of gene alterations of the four driver genes

In this study, we determined alterations in the *KRAS*, *TP53*, *CDKN2A*, and *SMAD4* genes using NGS, ddPCR, and IHC, as reported previously [30, 31]. The conclusive findings are presented in Supplemental Table S1.

### Evaluation of TILs and TLSs

In accordance with the method described previously [31], Tumor-infiltrating T cells (CD4^+^ cells, CD8^+^ cells, and FOXP3^+^ cells) were counted and classified into “high” and “low” density with a median division (S-Fig. 1a).

TLSs were assessed using H&E staining sections (S-Fig. 1b). TLSs within and around the tumor were defined as intratumoral and peritumoral TLSs, respectively. Cases with one or more TLSs were defined as TLSs “present,” and all cases were classified as “peritumoral and intratumoral,” “peritumoral,” and “absent” based on the presence and localization of TLS.

### Statistical analysis

JMP software version 12 (SAS Institute, Cary, NC, USA) and GraphPad Prism 8 (GraphPad Software, La Jolla, CA, USA) were used for statistical analyses. The significance of the results was determined using two-sided *P* values, where *P* values less than 0.05 were considered statistically significant. Overall survival (OS) was estimated by the Kaplan–Meier method and compared with the log-rank test and Cox proportional hazards model. The OS was defined as time between date of surgery and death. Patients who were alive at the end of follow-up were censored.

Binary categorical variables (high and low) were used as the outcome variables for TILs and the proportion of “cancer cell component,” “tumor-stromal collagen area,” and “α-SMA^+^ area.” Multivariable logistic regression was used to evaluate the association between the significant factors (alcohol consumption, smoking history, histological grade, *CDKN2A*/p16 alteration) and tumor-stromal collagen in PDAC. A Cox hazard model was used to evaluate the association between OS and tumor-stromal collagen in PDAC. A Cox hazard model was also used to evaluate the association between OS and tumor-stromal collagen in each adjuvant chemotherapy group. First, a stage-adjusted hazard ratio (HR) was calculated for each group and adjusted for the pathological stage. Multivariate HRs were then calculated for each chemotherapy group. A backward stepwise elimination with a threshold of *P = *0.05 was used to select variables in the multivariable model. The Chi-square test or Fisher's exact test was used to evaluate the association between categorical data. A t-test or analysis of variance assuming equal variances was performed to compare mean age and BMI.

## Results

### The low amount of tumor-stromal collagen in PDAC was significantly associated with poor differentiation and CDKN2A/p16 alteration

We examined the association between the amount of tumor-stromal collagen in PDAC and patient characteristics, including clinical and tumor-related information (Table 1). The patient characteristics in relation to the amount of tumor-stromal α-SMA^+^ myofibroblast and cancer cell components in PDAC are presented in Supplemental Tables S2 and S3, respectively.

**Table 1:** Clinical, pathological, and molecular characteristics of PDAC cases according to the amount of stromal collagen

Characteristics	All patients	Tumor-stromal collagen		*P* value
	(*N = *169)	Low (*N = *85)	High (*N = *84)	
Age (years), median (range)	69 (40–85)	69 (40–83)	69 (45–85)	0.47
Sex				0.32
Male	95 (56.2%)	51 (60.0%)	44 (52.4%)	
Female	74 (43.8%)	34 (40.0%)	40 (47.6%)	
BMI (kg/m^2^), median (range)	20.9 (14.3–33.2)	21.0 (14.3–33.2)	20.9 (15.0–32.3)	0.96
Family history of PDAC				0.98
Present	14 (8.3%)	7 (8.2%)	7 (8.3%)	
Absent	155 (91.7%)	78 (91.8%)	77 (91.7%)	
Alcohol consumption				0.05
< 50 (g/day)	151 (89.4%)	80 (94.1%)	71 (84.5%)	
≥ 50 (g/day)	18 (10.7%)	5 (5.9%)	13 (15.5%)	
Smoking history				0.009
Present	81 (47.9%)	32 (37.6%)	49 (58.3%)	
Absent	88 (52.1%)	53 (62.4%)	35 (41.7%)	
Diabetes mellitus				0.27
Present	65 (38.5%)	29 (34.1%)	36 (42.9%)	
Absent	104(61.5%)	56 (65.9%)	48 (57.1%)	
CA19-9				0.30
< 37 (U/ml)	40 (26.0%)	19 (22.4%)	25 (29.8%)	
≥ 37 (U/ml)	125 (74.0%)	66 (77.6%)	59 (70.2%)	
CEA				0.61
< 5 (ng/ml)	121 (71.6%)	59 (69.4%)	62 (73.8%)	
≥ 5 (ng/ml)	48 (28.4%)	26 (30.6%)	22 (26.2%)	
Pathological stage^a^				0.74
Ia/Ib	35 (20.7%)	18 (21.2%)	17 (20.2%)	
IIa/IIb	96 (56.8%)	50 (58.8%)	46 (54.8%)	
III	38 (22.5%)	17 (20.0%)	21 (25.0%)	
T factor				0.86
T1	25 (14.8%)	13 (15.2%)	12 (14.3%)	
T2	103 (60.9%)	52 (61.2%)	51 (60.7%)	
T3	35 (20.7%)	18 (21.2%)	17 (20.2%)	
T4	6 (3.6%)	2 (2.4%)	4 (4.8%)	
Tumor location				0.07
Head	120 (71.0%)	67 (78.8%)	53 (63.1%)	
Body	34 (20.1%)	12 (14.1%)	22 (26.2%)	
Tail	15 (8.9)	6 (7.1)	9 (10.7)	
Histological grade				0.01
Well/moderately differentiated	147 (87.0%)	68 (80.0%)	79 (94.0%)	
Poorly differentiated	22 (13.0%)	17 (20.0%)	5 (6.0%)	
Residual tumor status				0.39
R0	121 (71.6%)	58 (68.2%)	63 (75.0%)	
R1	48 (28.4%)	27 (31.8%)	21 (25.0%)	
R2	0 (0.0%)	0 (0.0%)	0 (0.0%)	
Neoadjuvant chemotherapy				0.81
Present	20 (11.8%)	11 (12.9%)	9 (10.7%)	
Absent	149 (88.2%)	74 (87.1%)	75 (89.3%)	
Adjuvant chemotherapy				0.50
S-1	78 (46.2%)	40 (47.1%)	38 (45.2%)	
GEM	43 (25.4%)	24 (28.2%)	19 (22.6%)	
None	48 (28.4%)	21 (24.7%)	27 (32.2%)	
*KRAS* mutation				0.77
Present	157 (92.9%)	78 (91.8%)	79 (94.0%)	
Absent	12 (7.1%)	7 (8.2%)	5 (6.0%)	
*TP53* alteration				0.41
Present	115 (68.1%)	55 (64.7%)	60 (71.4%)	
Absent	54 (31.9%)	30 (35.3%)	24 (28.6%)	
*CDKN2A*/p16 alteration				0.02
Present	109 (64.5%)	62 (72.9%)	47 (55.9%)	
Absent	60 (35.5%)	23 (27.1%)	37 (44.1%)	
*SMAD4* alteration				0.08
Present	68 (40.2%)	40 (47.1%)	28 (33.3%)	
Absent	101 (59.8%)	45 (52.9%)	56 (66.7%)	

Percentage (%) indicates the proportion of cases with a specific characteristic in all cases or strata of the amount of stromal collagen

^a^The pathological stage was diagnosed based on Union for International Cancer Control (UICC) TNM classification, 8th Edition

*PDAC* pancreatic ductal adenocarcinoma, *BMI* body mass index, *CA19-9* carbohydrate antigen 19–9, *CEA* carcinoembryonic antigen, *GEM* gemcitabine

A low amount of tumor-stromal collagen in PDAC was significantly associated with the absence of smoking history (*P = *0.009), poor differentiation (*P = *0.01), and the presence of *CDKN2A*/p16 alteration (*P = *0.02). No correlation was observed between the amount of tumor-stromal collagen and advanced cancer characteristics, such as pathological stage and T factor (Fig. 1g).

Multivariate analysis indicated that poor differentiation (multivariate OR = 3.82, 95% CI = 1.41–12.2, *P = *0.008) and *CDKN2A*/p16 alteration (multivariate OR = 2.06, 95% CI = 1.08–4.02, *P = *0.046) were independent predictors of a low tumor-stromal collagen amount in PDAC (Table 2).

**Table 2:** Logistic regression analyses of factors associated with the low amount of tumor-stromal collagen in PDAC

	Univariate		Multivariate	
	OR (95%CI)	*P* value	OR (95%CI)^a^	*P* value
Alcohol consumption		0.04		0.15
< 50 (g/day)	1		1	
≥ 50 (g/day)	0.34 (0.11–0.95)		0.44 (0.12–1.35)	
Smoking history		0.007		0.06
Absent	1		1	
Present	0.43 (0.23–0.79)		0.53 (0.27–1.03)	
Histological grade		0.005		0.008
Well/Moderately differentiated	1		1	
Poorly differentiated	3.95 (1.47–12.5)		3.82 (1.41–12.2)	
*CDKN2A*/p16 alteration		0.02		0.03
Absent	1		1	
Present	2.12 (1.12–4.08)		2.06 (1.08–4.02)	

^a^The model was adjusted for alcohol consumption, smoking history, histological grade, and *CDKN2A*/p16 alteration, selected variables

*PDAC* pancreatic ductal adenocarcinoma, *OR* odds ratio, *CI* confidence interval

### A low amount of tumor-stromal collagen in PDAC was an independent poor prognostic factor

**Fig. 2:**
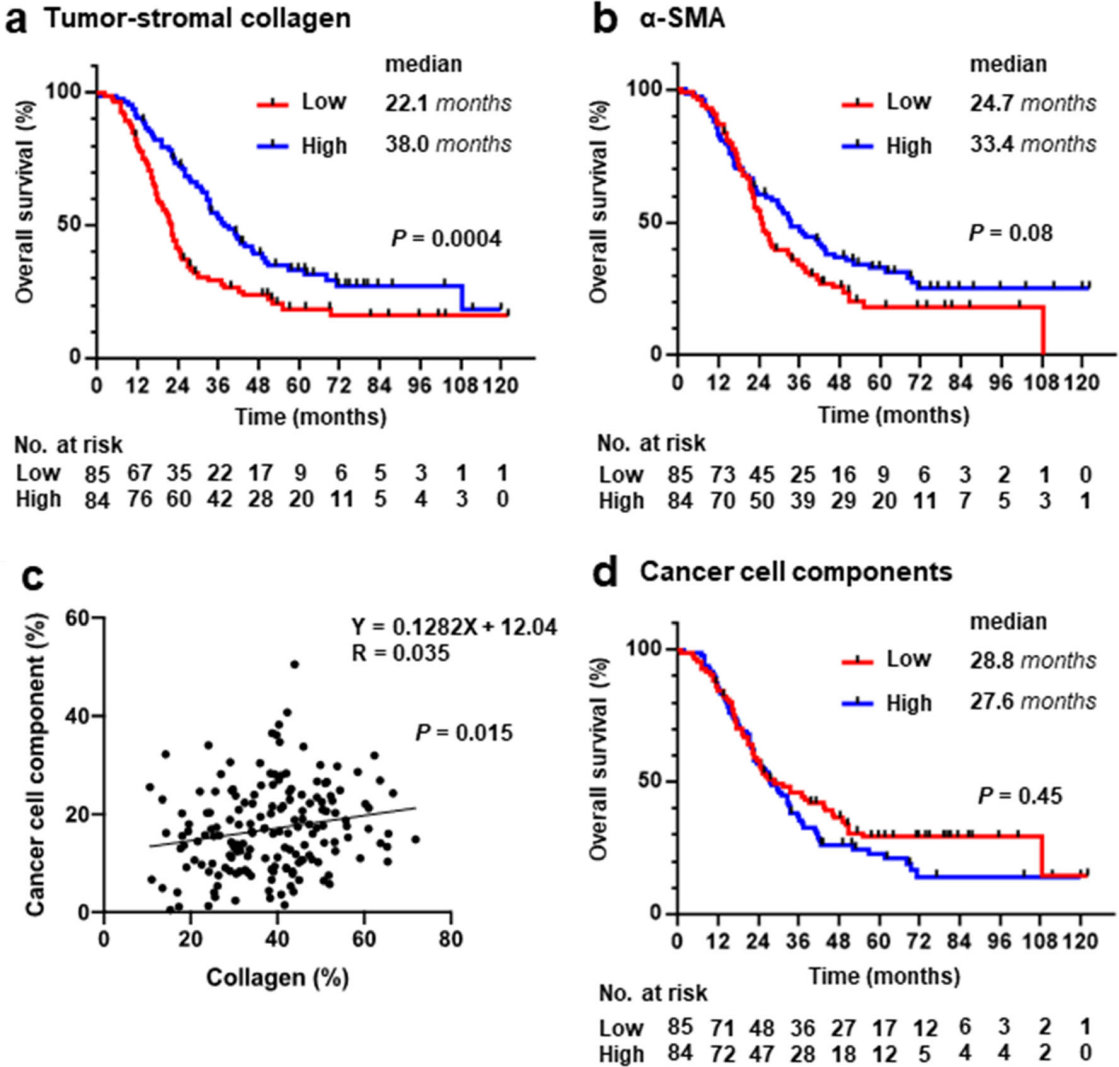
Overall survival (OS) of patients with pancreatic ductal adenocarcinoma according to the cancer cell component and fibrosis status of the tumor microenvironment. **a** Kaplan–Meier estimates of OS stratified by the proportion of tumor-stromal collagen, **b** Kaplan–Meier estimates of OS stratified by the proportion of tumor-stromal α-smooth muscle actin positive myofibroblasts, **c** The correlation between the proportion of tumor-stromal collagen and cancer cell component. **d** Kaplan–Meier estimates of OS stratified by the proportion of cancer cell components

We examined the association between the amount of tumor-stromal collagen and patient prognosis in PDAC. The patient cohort comprised 169 individuals diagnosed with PDAC, and the median follow-up duration was 26.8 (1.1–122.1) months. Patients with a low amount of tumor-stromal collagen had a significantly shorter OS than those with a high amount (22.1 months vs. 38.0 months, *P = *0.001) (Fig. 2a). α-SMA^+^ myofibroblast is the primary producer of tumor-stromal collagen in PDAC [34]. We also evaluated the association between amount of tumor-stromal α-SMA^+^ myofibroblast and patient prognosis and discovered a tendency for a similar association between the amount of tumor-stromal α-SMA^+^ myofibroblast and patient prognosis, with a shorter OS in patients with low amounts of tumor-stromal α-SMA^+^ myofibroblast compared to those with high amounts (24.7 months vs. 33.4 months, *P = *0.09) (Fig. 2b).

Using the univariate and multivariate Cox hazard model, we further analyzed the contribution of the tumor-stromal collagen amount in PDAC to OS (Table 3). Low levels of tumor-stromal collagen in PDAC were associated with poor patient prognosis, regardless of other characteristics, after adjusting for clinical characteristics (HR = 0.56, 95% CI = 0.39–0.81, *P = *0.002) and after adjusting for tumor characteristics (HR = 0.44, 95% CI = 0.29–0.65, *P < *0.0001). The low amount of tumor-stromal collagen in PDAC was also associated with poorly differentiated adenocarcinoma, a poor prognostic histological grade [35]. To eliminate the effect of the histological grade of PDAC on the prognosis, we analyzed 147 patients, excluding poorly differentiated adenocarcinoma. The low amount of tumor-stromal collagen in PDAC remained a poor prognostic factor after adjusting for clinical characteristics (HR = 0.48, 95% CI = 0.33–0.71, *P = *0.0002) and tumor characteristics (HR = 0.41, 95% CI = 0.27–0.62, *P < *0.0001).

**Table 3:** The association between overall survival and tumor-stromal collagen in PDAC

		Overall survival					
	No. of cases	Univariate		Multivariable		Multivariable	
		HR (95%CI)	*P* value	HR (95%CI)^a^	*P* value	HR (95%CI)^b^	*P* value
All cases	169						
Tumor-stromal collagen							
High	84	1		1		1	
Low	85	1.81 (1.26–2.61)	0.001	1.85 (1.27–2.72)	0.002	2.38 (1.59–3.56)	< 0.0001
Cases with well/moderately differentiated PDAC	147						
Tumor-stromal collagen							
High	79	1		1		1	
Low	68	1.99 (1.36–2.95)	0.0005	2.02 (1.46–3.34)	0.0002	2.39 (1.57–3.64)	< 0.0001

^a^HR was initially adjusted for clinical characteristics, including age, BMI, sex, family history of pancreatic cancer, alcohol consumption, smoking history, diabetes mellitus, CA19-9, and CEA

^b^HR was initially adjusted for tumor characteristics, including pathological stage, neoadjuvant chemotherapy, adjuvant chemotherapy, histological grade, residual tumor status, and gene alterations (*KRAS, TP53, CDKN2A*/p16, and *SMAD4*)

*PDAC* pancreatic ductal adenocarcinoma, *HR* hazard ratio, *CI* confidence interval

No correlation was discovered between the amount of tumor-stromal collagen and cancer cell component (Fig. 2c). In addition, the amount of cancer cell components was not associated with the prognosis of patients with PDAC (Fig. 2d).

These results demonstrated that a low amount of tumor-stromal collagen in PDAC was independently associated with poor patient prognosis.

### Low amount of tumor-stromal collagen correlated with poor prognosis in patients with PDAC who underwent adjuvant chemotherapy

Of the 169 patients with PDAC, 78 (46.2%), 43 (25.4%), and 48 (28.4%) underwent adjuvant chemotherapy with tegafur–gimeracil–oteracil potassium capsules (S-1), which is a 5-FU-based agent, gemcitabine (GEM), and none, respectively. Patient characteristics in relation to adjuvant chemotherapies are presented in Supplemental Table S4. Of the three groups, patients with PDAC who underwent S-1 (S-1 group), GEM (GEM group), and no (untreated group) adjuvant chemotherapies had significantly better outcomes in that order (*P = *0.0007) (S-Fig. 2a).

To explore the impact of tumor-stromal collagen amount on adjuvant chemotherapies, we stratified patients with PDAC by the amount of tumor-stromal collagen into high- and low-collagen groups and compared their prognoses for each adjuvant chemotherapy regimen (S-Figs. 2b–d). Patient characteristics in relation to the amount of tumor-stromal collagen in PDAC in the S-1, GEM, and untreated groups are presented in S-Tables S5, S6, and S7, respectively. In the S-1 group, OS was significantly shorter in the low-collagen group than in the high-collagen group (24.4 months vs. 57.0 months, *P = *0.0004) (S-Fig. 2b). However, in the GEM and untreated groups, there was no significant difference in OS between high- and low-collagen groups, although OS tended to be shorter in the low-collagen group (GEM group: 22.1 months vs. 35.5 months, *P = *0.17; untreated group: 17.1 months vs. 27.5 months, *P = *0.18) (S-Fig. 2c and d). In stage-adjusted and multivariate analyses, low amount of tumor-stromal collagen was significantly associated with poor prognosis in the S-1 and GEM groups (S-1 group: stage-adjusted HR = 2.86, 95% CI = 1.52–5.55, *P = *0.001; multivariable HR = 2.76, 95% CI = 1.36–5.79, *P = *0.005; GEM group: stage-adjusted HR = 2.90, 95% CI = 1.33–6.71, *P = *0.007; multivariate HR = 2.91, 95% CI = 1.34–6.71, *P = *0.007), whereas no significant difference was observed in the untreated group (stage-adjusted HR = 1.60, 95% CI = 0.82–3.09, *P = *0.17; multivariate HR = 1.50, 95% CI = 0.76–2.97, *P = *0.24) (Table 4).

**Table 4:** Effectiveness of adjuvant chemotherapy according to the amount of tumor stromal collagen in PDAC

	No. of cases	Overall survival					
		Univariate		Stage-adjusted		Multivariable	
		HR (95%CI)	*P* value	HR (95%CI)^a^	*P* value	HR (95%CI)	*P* value
S-1 group							
Tumor-stromal collagen							
High	38	1		1		1	
Low	40	2.69 (1.45–5.09)	0.002	2.86 (1.52–5.55)	0.001	2.76 (1.36–5.79)^b^	0.005
GEM group							
Tumor-stromal collagen							
High	19	1		1		1	
Low	24	1.49 (0.78–2.91)	0.23	2.90 (1.33–6.71)	0.007	2.91 (1.34–6.71)^c^	0.007
Untreated group							
Tumor-stromal collagen							
High	27	1		1		1	
Low	21	1.50 (0.78–2.86)	0.22	1.60 (0.82–3.09)	0.17	1.50 (0.76–2.97)^d^	0.24

^a^The stage-adjusted HR was adjusted for the pathological stage

^b^HR was initially adjusted for alcohol consumption, smoking history, diabetes mellitus, CA19-9, tumor location, histological grade, and *CDKN2A*/p16 alteration

^c^HR was initially adjusted for pathological stage and *SMAD4* alteration

^d^HR was initially adjusted for diabetes mellitus and tumor location

*PDAC* pancreatic ductal adenocarcinoma, *HR* hazard ratio, *CI* confidence interval

These results suggest that the amount of tumor-stromal collagen in PDAC may influence chemotherapy sensitivity.

### The amount of tumor-stromal collagen in PDAC was associated with CD4^+^ and CD8^+^ TILs recruitment and TLSs formation

We analyzed the relationship between tumor-stromal collagen, tumor-infiltrating immune cell populations, and patient survival in PDAC (Table 5). The number of CD4^+^ and CD8^+^ TILs tended to be small in the low-collagen group compared to the high-collagen group (CD4^+^ TILs: *P = *0.046; CD8^+^ TILs: *P = *0.09).

**Table 5:** The association between immune status and tumor-stromal collagen in PDAC

Variables	No. of cases	Tumor-stromal collagen		*P* value
All cases	(*N = *169)	Low (*N = *85)	High (*N = *84)	
CD4^+^ TILs				0.046
High	85 (50.3%)	36 (42.4%)	49 (58.3%)	
Low	84 (49.7%)	49 (57.6%)	35 (41.7%)	
CD8^+^ TILs				0.09
High	85 (50.3%)	37 (43.5%)	48 (57.1%)	
Low	84 (49.7%)	48 (56.5%)	36 (42.9%)	
FOXP3^+^ TILs				0.99
High	85 (50.3%)	43 (50.6%)	42 (50.0%)	
Low	84 (49.7%)	42 (49.4%)	42 (50.0%)	
TLSs				0.001
Peritumoral + Intratumoral	27 (16.0%)	11 (12.9%)	16 (19.0%)	
Peritumoral	90 (53.3%)	35 (41.2%)	55 (65.5%)	
Absent	52 (30.8%)	39 (45.9%)	13 (15.5%)	

Cases with well/moderately differentiated PDAC	(*N = *147)	Low (*N = *68)	High (*N = *79)	
CD4^+^ TILs				0.002
High	71 (48.3%)	23 (33.8%)	48 (60.8%)	
Low	76 (51.7%)	45 (66.2%)	31 (39.2%)	
CD8^+^ TILs				0.10
High	76 (51.7%)	30 (44.1%)	46 (58.2%)	
Low	71 (48.3%)	38 (55.9%)	33 (41.8%)	
FOXP3^+^ TILs				0.99
High	72 (49.0%)	33 (48.5%)	39 (49.4%)	
Low	75 (51.0%)	35 (51.5%)	40 (50.6%)	
TLSs				0.001
Peritumoral + Intratumoral	22 (15.0%)	8 (11.8%)	14 (17.7%)	
Peritumoral	80 (54.4%)	27 (39.7%)	53 (67.1%)	
Absent	45 (30.6%)	33 (48.5%)	12 (15.2%)	

*PDAC* pancreatic ductal adenocarcinoma, *TILs* tumor-infiltrating T cells, *TLSs* tertiary lymphoid structures

TLSs have been recently speculated to contribute to the strength of tumor-specific immune responses [25]. We next examined the relationship between the amount of tumor-stromal collagen and the presence and location of TLSs in PDAC. Of the 169 patients with PDAC, 27 (16.0%), 90 (53.3%), and 52 (30.8%) had peritumoral plus intratumoral, peritumoral, and absent TLSs, respectively. All patients with PDAC with intratumoral TLSs had peritumoral TLSs. TLSs, including peritumoral and intratumoral, were significantly fewer in the low-collagen group than in the high-collagen group (*P = *0.001). Poor tumor grade is reportedly associated with increased FOXP3^+^ TILs and decreased CD8^+^ TILs in PDAC [36]. We re-analyzed 147 patients, excluding poorly differentiated adenocarcinoma, and obtained similar results.

These results suggest that the amount of tumor-stromal collagen in PDAC may be related to the immune status of PDAC.

## Discussion

This study presents several noteworthy findings about the TME of human PDAC. First, tumor-stromal collagen was an independent prognostic factor. Second, tumor-stromal collagen influenced the efficacy of adjuvant chemotherapy. Third, the amount of tumor-stromal collagen was significantly linked with the histological grade and the frequency of *CDKN2A*/p16 alteration. Despite the growing recognition of the impact of tumor-stromal collagen on the molecular and immunological characteristics of PDAC, a comprehensive understanding of its role in tumor progression and chemotherapy sensitivity remains elusive, particularly in the Japanese population. Our findings suggest that tumor-stromal collagen may play a crucial role in the progression and chemotherapy sensitivity of PDAC in close association with anti-tumor immune responses.

We observed a decrease in the number of CD4^+^ and CD8^+^ TILs and TLSs formation, along with poor patient prognosis, in PDAC with low tumor-stromal collagen. Conversely, we did not find a significant association between the amount of cancer cell components and patient prognosis. TILs and TLSs are crucial components of the anti-tumor immune system and have been correlated with a more favorable prognosis in PDAC [27, 28, 37]. The results of previous studies utilizing genetically engineered mouse models [11] and our examination of human PDAC specimens suggested that a decrease in tumor-stromal collagen in PDAC negatively affected the anti-immune system, such as CD4^+^ and CD8^+^ TILs. Furthermore, the presence of TLS has also been demonstrated to correlate with an increased number of CD8^+^ TILs in human PDAC [38]. Our findings and those of previous studies indicate that a decreased amount of tumor-stromal collagen in PDAC is strongly linked to the anti-tumor immune system, resulting in poorer patient prognosis. Furthermore, our results imply that the patient prognosis in PDAC could be more significantly influenced by the tumor characteristics that determine the composition of the TME, such as tumor-stromal collagen and immune cells, rather than the amount of cancer cells themselves.

We also revealed a significant association between a high amount of tumor-stromal collagen and improved prognosis in patients with PDAC who underwent adjuvant chemotherapy. Previous studies have indicated that increased tumor stroma impaired chemotherapy sensitivity by creating a hypoxic environment and hindering drug delivery, leading to poor patient prognosis [39–42]. In contrast, recent studies have indicated that increased tumor stroma correlated with a high number of CD4^+^ and CD8^+^ TILs, leading to better patient prognosis [11]. 5-Fluorouracil (5-FU) and GEM have been demonstrated to enhance anti-tumor immunity by increasing tumor-associated antigens and interferon-gamma (IFN-γ) production while reducing regulatory T cells, myeloid-derived suppressor cells, and immunosuppressive cytokines, including transforming growth factor β (TGFβ) [43, 44]. Thus, 5-FU and GEM are closely linked to the immune environment. Our findings, in agreement with previous studies, suggest that S-1, a prodrug of 5-FU, and GEM may be more effective by enhancing tumor immunity in PDAC patients with a high amount of tumor-stromal collagen, that is, abundant tumor-infiltrating effector T cells.

We have revealed an intriguing connection between PDACs with low tumor-stromal collagen and the variant allele frequency of *CDKN2A*/p16. Additionally, patients with low tumor-stromal collagen exhibited worse prognosis and high occurrence of poorly differentiated adenocarcinoma. However, no correlation was observed between the tumor stromal collagen amount and the pathological stage. Previous studies using various PDAC mouse models have demonstrated that inhibiting fibrotic ECM formation increases the frequency of undifferentiated tumors [11, 17, 18]. The correlation between fibrotic ECM and the histological grade of PDAC has also been established in human specimens through IHC [17, 45]. However, the relationship between driver gene alterations, including *CDKN2A*/p16, and fibrotic ECM in PDAC has not been previously explored. Several previous studies have reported Several previous studies have reported the mechanism by which *CDKN2A*/p16 regulates fibrosis in various organs. For instance, in the kidney, Wnt9a promotes renal fibrosis by activating β-catenin signaling. This process is associated with the cellular senescence of the renal tubule and secretion of the senescence-associated secretory phenotype (SASP); moreover, fibrosis is reportedly inhibited by p16 knockdown [46]. In the lung, increased p16 expression and senescence-associated β-galactosidase activity was observed in the lung epithelial cells of patients with idiopathic pulmonary fibrosis [47]. The secretion of SASP factors by primary fibrotic mouse alveolar epithelial type II cells has also been confirmed in vitro [47]. In the thyroid, the severity of fibrosis is significantly low in p16-negative papillary thyroid carcinoma compared to p16-positive papillary thyroid carcinoma [48]. These reports suggested that p16 expression is involved in the formation of fibrosis, supporting our present data. However, it is important to note that the relationship between *CDKN2A*/p16 and fibrosis can vary among organs. For example, in liver cirrhosis, p16 expression is decreased in activated hepatic stellate cells (HSC) in mice and humans. Drug-induced liver fibrosis was reportedly more severe in *CDKN2A*/p16 knockout mice than wild-type mice, and *CDKN2A*/p16 deficiency promotes the production of reactive oxygen species in HSC via p38 MAPK signaling [49]. Thus, given the diverse nature of the relationship between *CDKN2A*/p16 and fibrosis in different tissues, and the lack of definitive consensus, investigating this relationship in the pancreas, including pancreatic cancer, is essential. This study suggests that *CDKN2A*/p16 alteration may contribute to the low fibrotic ECM formation, independent of tumor progression, in PDAC. Further research, including mouse models, is necessary to elucidate the causal relationship and underlying mechanisms between *CDKN2A*/p16 and fibrosis in PDAC.

The strength of this study is that we analyzed and quantified cancer cell components and tumor stroma using a whole section, unlike previous studies, in which a part of the tumor was analyzed using magnified images. Our method allowed more accurate and quantitative evaluations of the entire tumor. Furthermore, the utilization of EVG staining for assessing collagen is a well-established method in clinical practice, and our findings can be readily translated to real-world patient care. Profiling tumor-stromal collagen in surgically resected specimens holds promise as a biomarker for predicting the prognosis and chemotherapy effectiveness. Despite the advantages of our study design, this study had several limitations. First, the sample size was limited to only surgically treated cases, and it remains unclear whether the results apply to non-resected cases. Second, only a representative section with the largest tumor area was analyzed, which may not completely reflect the heterogeneity of PDAC. However, our method enabled a more accurate evaluation of the collagen amount than previous studies. Finally, the selection of postoperative chemotherapy was at the physicians' discretion, potentially introducing selection bias.

### Supplementary Information

Below is the link to the electronic supplementary material.

Supplementary file1 (PDF 7495 KB)

Supplementary file2 (PDF 251 KB)

Supplementary file3 (DOCX 93 KB)
