# Supplementary material for: A comprehensive analysis of tumor-stromal collagen in relation to pathological, molecular, and immune characteristics and patient survival in pancreatic ductal adenocarcinoma
Source: J Gastroenterol. 2023 Jul 21;58(10):1055–67. doi: 10.1007/s00535-023-02020-8 (PMC10522520; doi:10.1007/s00535-023-02020-8)
Supplement: Supplementary file 1 — Supplementary file1 (PDF 7495 KB) [file 535_2023_2020_MOESM1_ESM.pdf]

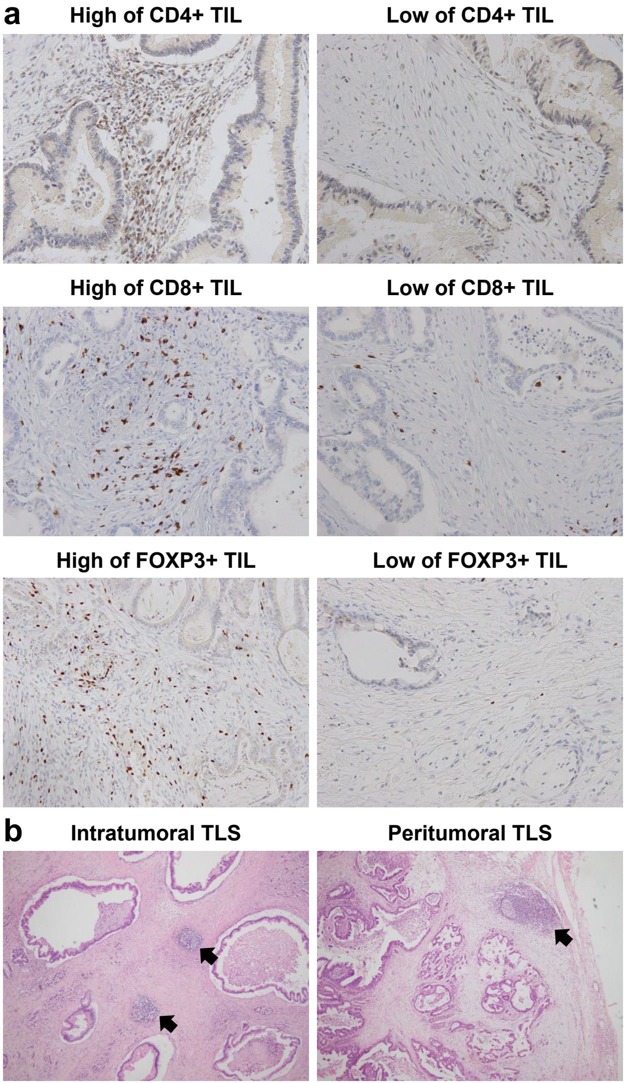
a
High of CD4+ TIL
Low of CD4+ TIL
High of CD8+ TIL
Low of CD8+ TIL
High of FOXP3+ TIL
Low of FOXP3+ TIL
b
Intratumoral TLS
Peritumoral TLS
