# Supplementary figures and images for: A comprehensive analysis of tumor-stromal collagen in relation to pathological, molecular, and immune characteristics and patient survival in pancreatic ductal adenocarcinoma
Source: J Gastroenterol. 2023 Jul 21;58(10):1055–67. doi: 10.1007/s00535-023-02020-8 (PMC10522520; doi:10.1007/s00535-023-02020-8)

**a**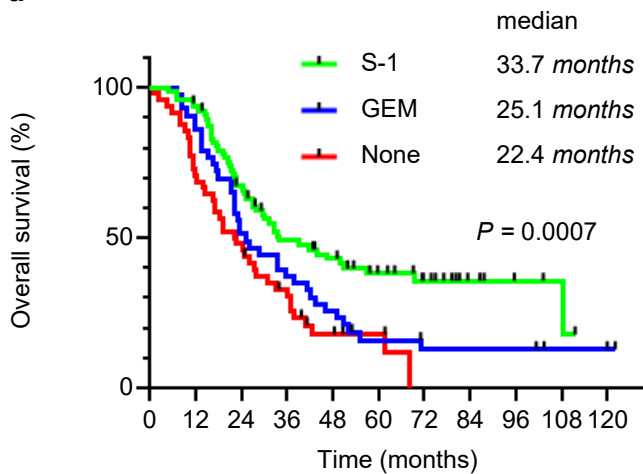

No. at risk

|           |    |    |    |    |    |    |    |   |   |   |   |
|-----------|----|----|----|----|----|----|----|---|---|---|---|
| S-1       | 78 | 72 | 50 | 34 | 28 | 19 | 13 | 6 | 3 | 2 | 0 |
| GEM       | 43 | 37 | 22 | 16 | 11 | 6  | 4  | 4 | 4 | 2 | 1 |
| Untreated | 48 | 34 | 23 | 14 | 6  | 4  | 0  | 0 | 0 | 0 | 0 |

**b**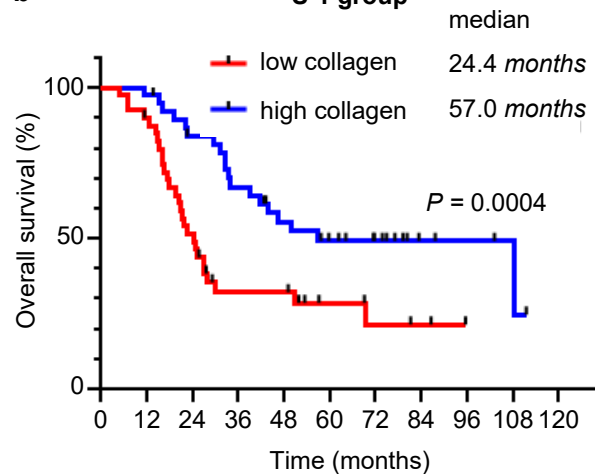

No. at risk

|      |    |    |    |    |    |    |    |   |   |   |   |
|------|----|----|----|----|----|----|----|---|---|---|---|
| Low  | 40 | 35 | 20 | 10 | 10 | 5  | 3  | 2 | 0 | 0 | 0 |
| High | 38 | 37 | 30 | 24 | 18 | 14 | 10 | 4 | 3 | 2 | 0 |

**c**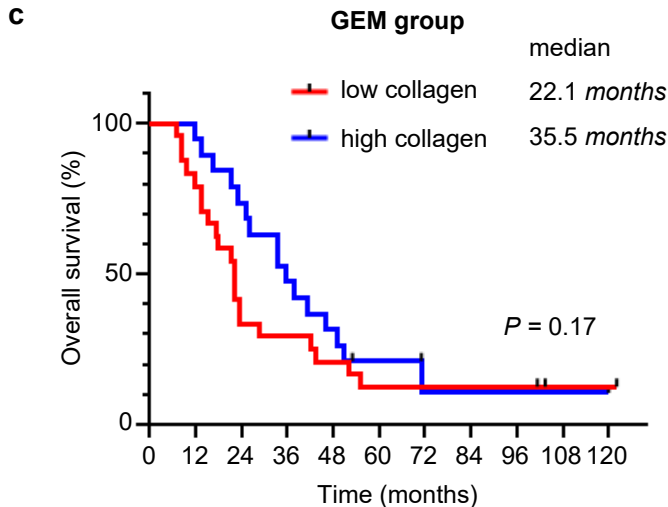

No. at risk

|      |    |    |    |   |   |   |   |   |   |   |   |
|------|----|----|----|---|---|---|---|---|---|---|---|
| Low  | 24 | 19 | 8  | 7 | 5 | 3 | 3 | 3 | 3 | 1 | 1 |
| High | 19 | 18 | 14 | 9 | 6 | 3 | 1 | 1 | 1 | 1 | 0 |

**d**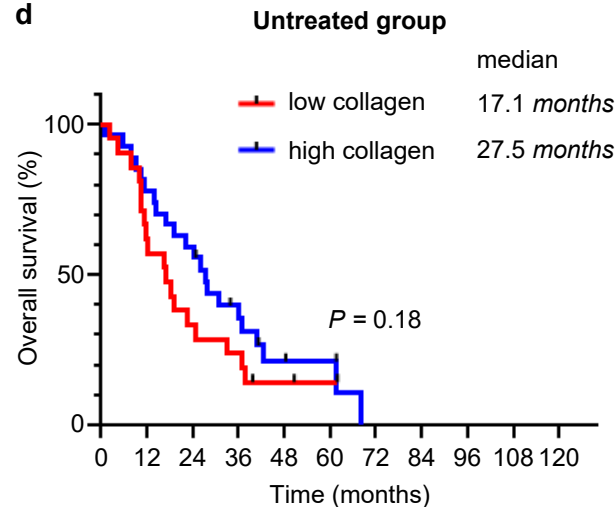

No. at risk

|      |    |    |    |   |   |   |   |   |   |   |   |
|------|----|----|----|---|---|---|---|---|---|---|---|
| Low  | 21 | 13 | 7  | 5 | 2 | 1 | 0 | 0 | 0 | 0 | 0 |
| High | 27 | 21 | 16 | 9 | 4 | 3 | 0 | 0 | 0 | 0 | 0 |

Supplement: Supplementary file 2 — Supplementary file2 (PDF 251 KB) [file 535_2023_2020_MOESM2_ESM.pdf]
