# Supplementary material for: A comprehensive analysis of tumor-stromal collagen in relation to pathological, molecular, and immune characteristics and patient survival in pancreatic ductal adenocarcinoma
Source: J Gastroenterol. 2023 Jul 21;58(10):1055–67. doi: 10.1007/s00535-023-02020-8 (PMC10522520; doi:10.1007/s00535-023-02020-8)
Supplement: Supplementary file 3 — Supplementary file3 (DOCX 93 KB) [file 535_2023_2020_MOESM3_ESM.docx]

**Supplemental Table S1.** Integrated classification of *TP53*, *CDKN2A/*p16 and *SMAD4* by

next-generation sequencing (NGS), droplet digital PCR (ddPCR) and immunohistochemistry

| Gene^*^ |  | | Molecular alteration^#^ | |
| --- | --- | --- | --- | --- |
|  |  | | Wild | Altered |
| *TP53* | IHC | Wild type | 45 | 0 |
|  |  | Overexpression | 9 | 81 |
|  |  | Loss | 16 | 18 |
| *CDKN2A/*p16 | IHC | Wild type | 55 | 5 |
|  |  | Loss | 31 | 78 |
| *SMAD4* | IHC | Wild type | 89 | 12 |
|  |  | Loss | 10 | 58 |

^*^For *TP53*, *CDKN2A/*p16 and *SMAD4,* the final classification of gene alterations was analyzed by comprehensively

considering the NGS data, ddPCR data and the IHC expression pattern.

^#^Molecular alteration includes SNV or short indels or CNV.

Red font indicates cases with presence of final integrated gene alterations.

PCR, polymerase chain reaction; IHC, immunohistochemistry; CNV, copy number variation.

**Supplemental Table S2.** Patient characteristics in relation to the amount of tumor-stromal α-SMA^+^ myofibroblast in PDAC

| **Characteristics** | **All patients** | **Tumor-stromal**  **α-SMA^+^ myofibroblast** | | ***P* value** |
| --- | --- | --- | --- | --- |
|  | (N = 169) | Low (N = 85) | High (N = 84) |  |
| Age (years), median (range) | 69 (40-85) | 70 (40-85) | 69 (45-84) | 0.75 |
| Sex |  |  |  | 0.99 |
| Male | 95 (56.2%) | 48 (56.5%) | 47 (55.9%) |  |
| Female | 74 (43.8%) | 37 (43.5%) | 37 (44.1%) |  |
| BMI (kg/m^2^), median (range) | 20.9 (14.3-33.2) | 20.9 (14.3-33.2) | 21.0 (16.5-32.3) | 0.09 |
| Family history of PDAC |  |  |  | 0.78 |
| Present | 14 (8.3%) | 8 (9.4%) | 6 (7.1%) |  |
| Absent | 155 (91.7%) | 77 (90.6%) | 78 (92.9%) |  |
| Alcohol consumption |  |  |  | 0.80 |
| <50 (g/day) | 151 (89.4%) | 75 (88.2%) | 76 (90.5%) |  |
| ≥50 (g/day) | 18 (10.6%) | 10 (11.8%) | 8 (9.5%) |  |
| Smoking history |  |  |  | 0.05 |
| Present | 81 (47.9%) | 34 (40.0%) | 47 (55.9%) |  |
| Absent | 88 (52.1%) | 51 (60.0%) | 37 (44.1%) |  |
| Diabetes mellitus |  |  |  | 0.16 |
| Present | 65 (38.5%) | 28 (32.9%) | 37 (44.1%) |  |
| Absent | 104 (61.5%) | 57 (67.1%) | 47 (55.9%) |  |
| CA19-9 |  |  |  | 0.03 |
| <37 (U/ml) | 44 (26.0%) | 16 (18.8%) | 28 (33.3%) |  |
| ≥37 (U/ml) | 125 (74.0%) | 69 (81.2%) | 56 (66.7%) |  |
| CEA |  |  |  | 0.50 |
| <5 (ng/ml) | 121 (71.6%) | 63 (74.1%) | 58 (69.1%) |  |
| ≥5 (ng/ml) | 48 (28.4%) | 22 (25.9%) | 26 (30.9%) |  |
| Pathological stage^a^ |  |  |  | 0.82 |
| Ia/Ib | 35 (20.7%) | 16 (18.8%) | 19 (22.6%) |  |
| IIa/IIb | 96 (56.8%) | 49 (57.7%) | 47 (55.9%) |  |
| III | 38 (22.5%) | 20 (23.5%) | 18 (21.4%) |  |
| T factor |  |  |  |  |
| T1 | 25 (14.8%) | 15 (17.7%) | 10 (11.9%) | 0.047 |
| T2 | 103 (60.9%) | 49 (57.6%) | 54 (64.3%) |  |
| T3 | 35 (20.7%) | 15 (17.6%) | 20 (23.8%) |  |
| T4 | 6 (3.6%) | 6 (7.1%) | 0 (0%) |  |
| Tumor location |  |  |  | 0.44 |
| Head | 120 (71.0%) | 64 (75.3%) | 56 (66.7%) |  |
| Body | 34 (20.1%) | 14 (16.5%) | 20 (23.8%) |  |
| Tail | 15 (8.9%) | 7 (8.2%) | 8 (9.5%) |  |
| Histological grade |  |  |  | 0.49 |
| Well/moderately differentiated | 147 (87.0%) | 72 (84.7%) | 75 (89.3%) |  |
| Poorly differentiated | 22 (13.0%) | 13 (15.3%) | 9 (10.7%) |  |
| Residual tumor status |  |  |  | 0.50 |
| R0 | 121 (71.6%) | 63 (74.1%) | 58 (69.1%) |  |
| R1 | 48 (28.4%) | 22 (25.9%) | 26 (30.9%) |  |
| R2 | 0 (0.0%) | 0 (0.0%) | 0 (0.0%) |  |
| Neoadjuvant chemotherapy |  |  |  | 0.99 |
| Present | 20 (11.8%) | 10 (11.8%) | 10 (11.9%) |  |
| Absent | 149 (88.2%) | 75 (88.2%) | 74 (88.1%) |  |
| Adjuvant chemotherapy |  |  |  | 0.06 |
| S-1 | 78 (46.1%) | 33 (38.8%) | 45 (53.6%) |  |
| GEM | 43 (25.4%) | 28 (32.9%) | 15 (17.8%) |  |
| None | 48 (28.4%) | 24 (28.2%) | 24 (28.6%) |  |
| *KRAS* mutation |  |  |  | 0.57 |
| Present | 157 (92.9%) | 80 (94.1%) | 77 (91.7%) |  |
| Absent | 12 (7.1%) | 5 (5.9%) | 7 (8.3%) |  |
| *TP53* alteration |  |  |  | 0.99 |
| Present | 115 (68.1%) | 58 (68.2%) | 57 (67.9%) |  |
| Absent | 54 (31.9%) | 27 (31.8%) | 27 (32.1%) |  |
| *CDKN2A*/p16 alteration |  |  |  | 0.99 |
| Present | 109 (64.5%) | 55 (64.7%) | 54 (64.3%) |  |
| Absent | 60 (35.5%) | 30 (35.3%) | 30 (35.7%) |  |
| *SMAD4* alteration |  |  |  | 0.21 |
| Present | 68 (40.2%) | 30 (35.3%) | 38 (45.2%) |  |
| Absent | 101 (59.8%) | 55 (64.7%) | 46 (54.8%) |  |

Percentage (%) indicates the proportion of cases with a specific characteristic in all cases or strata of the amount of stromal collagen.

^a^The pathological stage was diagnosed based on Union for International Cancer Control (UICC) TNM classification, 8th Edition.

BMI, body mass index; CA19-9, carbohydrate antigen 19-9; CEA, carcinoembryonic antigen; GEM, gemcitabine; PDAC, pancreatic ductal adenocarcinoma.

**Supplemental Table S3.** Patient characteristics in relation to the amount of cancer cell component in PDAC

| **Characteristics** | **All patients** | **Cancer cell components** | | ***P* value** |
| --- | --- | --- | --- | --- |
|  | (N=169) | Low (N=85) | High (N=84) |  |
| Age (years), median (range) | 69 (40-85) | 69 (40-83) | 70 (45-85) | 0.35 |
| Sex |  |  |  | 0.54 |
| Male | 95 (56.2%) | 50 (58.8%) | 45 (53.6%) |  |
| Female | 74 (43.8%) | 35 (41.2%) | 39 (46.4%) |  |
| BMI (kg/m^2^), median (range) | 20.9 (14.3-33.2) | 21.0 (15.6-32.3) | 20.9 (14.3-33.2) | 0.21 |
| Family history of PDAC |  |  |  | 0.78 |
| Present | 14 (8.3%) | 8 (9.4%) | 6 (7.1%) |  |
| Absent | 155 (91.7%) | 77 (90.6%) | 78 (92.9%) |  |
| Alcohol consumption |  |  |  | 0.80 |
| <50 (g/day) | 151 (89.4%) | 75 (88.2%) | 76 (90.5%) |  |
| ≥50 (g/day) | 18 (10.6%) | 10 (11.8%) | 8 (9.5%) |  |
| Smoking history |  |  |  | 0.88 |
| Present | 81 (47.9%) | 40 (47.1%) | 41 (48.8%) |  |
| Absent | 88 (52.1%) | 45 (52.9%) | 43 (51.2%) |  |
| Diabetes mellitus |  |  |  | 0.15 |
| Present | 65 (38.5%) | 28 (32.9%) | 37 (44.1%) |  |
| Absent | 104 (61.5%) | 57 (67.1%) | 47 (55.9%) |  |
| CA19-9 |  |  |  | 0.86 |
| <37 (U/ml) | 44 (26.0%) | 23 (27.1%) | 21 (25.0%) |  |
| ≥37 (U/ml) | 125 (74.0%) | 62 (72.9%) | 63 (75.0%) |  |
| CEA |  |  |  | 0.04 |
| <5 (ng/ml) | 121 (71.6%) | 67 (78.8%) | 54 (64.3%) |  |
| ≥5 (ng/ml) | 48 (28.4%) | 18 (21.2%) | 30 (35.7%) |  |
| Pathological stage^a^ |  |  |  | 0.65 |
| Ⅰa/Ⅰb | 35 (20.7%) | 20 (23.5%) | 15 (17.9%) |  |
| Ⅱa/Ⅱb | 96 (56.8%) | 46 (54.1%) | 50 (59.5%) |  |
| Ⅲ | 38 (22.5%) | 19 (22.4%) | 19 (22.6%) |  |
| T factor |  |  |  | 0.35 |
| T1 | 25 (14.8%) | 14 (16.5%) | 11 (13.1%) |  |
| T2 | 103 (60.9%) | 49 (57.6%) | 54 (64.3%) |  |
| T3 | 35 (20.7%) | 17 (20.0%) | 18 (21.4%) |  |
| T4 | 6 (3.6%) | 5 (5.9%) | 1 (1.2%) |  |
| Tumor location |  |  |  | 0.19 |
| Head | 120 (71.0%) | 55 (64.7%) | 65 (77.4%) |  |
| Body | 34 (20.1%) | 21 (24.7%) | 13 (15.5%) |  |
| Tail | 15 (8.9%) | 9 (10.6%) | 6 (7.1%) |  |
| Histological grade |  |  |  | 0.01 |
| Well/moderate differentiated | 147 (87.0%) | 68 (80.0%) | 79 (94.1%) |  |
| Poorly differentiated | 22 (13.0%) | 17 (20.0%) | 5 (5.9%) |  |
| Residual tumor status |  |  |  | 0.86 |
| R0 | 121 (71.6%) | 60 (70.6%) | 61 (72.6%) |  |
| R1 | 48 (28.4%) | 25 (29.4%) | 23 (27.4%) |  |
| R2 | 0 (0.0%) | 0 (0.0%) | 0 (0.0%) |  |
| Neoadjuvant chemotherapy |  |  |  | 0.09 |
| Present | 20 (11.8%) | 14 (16.5%) | 6 (7.1%) |  |
| Absent | 149 (88.2%) | 71 (83.5%) | 78 (92.9%) |  |
| Adjuvant chemotherapy |  |  |  | 0.34 |
| S-1 | 78 (46.1%) | 43 (50.6%) | 35 (41.7%) |  |
| GEM | 43 (25.4%) | 22 (25.9%) | 21 (25.0%) |  |
| None | 48 (28.4%) | 20 (23.5%) | 28 (33.3%) |  |
| *KRAS* mutation |  |  |  | 0.77 |
| Present | 157 (92.9%) | 78 (91.8%) | 79 (94.1%) |  |
| Absent | 12 (7.1%) | 7 (8.2%) | 5 (5.9%) |  |
| *TP53* alteration |  |  |  | 0.62 |
| Present | 115 (68.1%) | 56 (65.9%) | 59 (70.2%) |  |
| Absent | 54 (31.9%) | 29 (34.1%) | 25 (29.8%) |  |
| *CDKN2A*/p16 alteration |  |  |  | 0.87 |
| Present | 109 (64.5%) | 54 (63.5%) | 55 (65.5%) |  |
| Absent | 60 (35.5%) | 31 (36.5%) | 29 (34.5%) |  |
| *SMAD4* alteration |  |  |  | 0.53 |
| Present | 68 (40.2%) | 32 (37.6%) | 36 (42.9%) |  |
| Absent | 101 (59.8%) | 53 (62.4%) | 48 (57.1%) |  |

Percentage (%) indicates the proportion of cases with a specific characteristic in all cases or strata of the amount of stromal collagen.

^a^The pathological stage was diagnosed based on Union for International Cancer Control (UICC) TNM classification, 8th Edition.

BMI, body mass index; CA19-9, carbohydrate antigen 19-9; CEA, carcinoembryonic antigen; GEM, gemcitabine; PDAC, pancreatic ductal adenocarcinoma.

**Supplemental Table S4.** Patient characteristics in relation to adjuvant chemotherapies in PDAC

| **Characteristics** | **Adjuvant chemotherapy** | | | ***P* value** |
| --- | --- | --- | --- | --- |
|  | **None** | **S-1** | **GEM** |  |
|  | (N = 48) | (N = 78) | (N = 43) |  |
| Age (years), median (range) | 70 (50-84) | 69 (40-82) | 70 (45-85) | 0.006 |
| Sex |  |  |  | 0.43 |
| Male | 25 (52.1%) | 48 (61.5%) | 22 (51.2%) |  |
| Female | 23 (47.9%) | 30 (38.5%) | 21 (48.8%) |  |
| BMI (kg/m^2^), median (range) | 20.9 (16.1-27.6) | 20.9 (14.3-33.2) | 20.8 (14.9-31.2) | 0.03 |
| Family history of PDAC |  |  |  | 0.30 |
| Present | 3 (6.3%) | 5 (6.4%) | 6 (14.0%) |  |
| Absent | 45 (93.7%) | 73 (93.6%) | 37 (86.0%) |  |
| Alcohol consumption |  |  |  | 0.09 |
| <50 (g/day) | 39 (81.2%) | 73 (93.6%) | 39 (90.7%) |  |
| ≥50 (g/day) | 9 (18.8%) | 5 (6.4%) | 4 (9.3%) |  |
| Smoking history |  |  |  | 0.007 |
| Present | 24 (50.0%) | 45 (57.7%) | 12 (27.9%) |  |
| Absent | 24 (50.0%) | 33 (42.3%) | 31 (72.1%) |  |
| Diabetes mellitus |  |  |  | 0.43 |
| Present | 20 (41.7%) | 32 (41.0%) | 13 (30.2%) |  |
| Absent | 28 (58.3%) | 46 (59.0%) | 30 (69.8%) |  |
| CA19-9 |  |  |  | 0.76 |
| <37 (U/ml) | 12 (25.0%) | 19 (24.4%) | 13 (30.2%) |  |
| ≥37 (U/ml) | 36 (75.0%) | 59 (75.6%) | 30 (69.8%) |  |
| CEA |  |  |  | 0.31 |
| <5 (ng/ml) | 31 (64.6%) | 56 (71.8%) | 34 (79.1%) |  |
| ≥5 (ng/ml) | 17 (35.4%) | 22 (28.2%) | 9 (20.9%) |  |
| Pathological stage^a^ |  |  |  | 0.67 |
| Ia/Ib | 8 (16.7%) | 19 (24.4%) | 8 (18.6%) |  |
| IIa/IIb | 31 (64.6%) | 40 (51.3%) | 25 (58.1%) |  |
| III | 9 (18.7%) | 19 (24.4%) | 10 (23.3%) |  |
| T factor |  |  |  | 0.15 |
| T1 | 6 (12.5%) | 14 (17.9%) | 5 (11.6%) |  |
| T2 | 27 (56.3%) | 48 (61.5%) | 28 (65.1%) |  |
| T3 | 14 (29.2%) | 15 (19.2%) | 6 (14.0%) |  |
| T4 | 1 (2.1%) | 1 (1.3%) | 4 (9.3%) |  |
| Tumor location |  |  |  | 0.14 |
| Head | 38 (79.2%) | 49 (62.8%) | 33 (76.7%) |  |
| Body | 7 (14.6%) | 22 (28.2%) | 5 (11.6%) |  |
| Tail | 3 (6.3%) | 7 (9.0%) | 5 (11.6%) |  |
| Histological grade |  |  |  | 0.51 |
| Well/moderately differentiated | 44 (91.7%) | 66 (84.6%) | 37 (86.1%) |  |
| Poorly differentiated | 4 (8.3%) | 12 (15.4%) | 6 (13.9%) |  |
| Residual tumor status |  |  |  | 0.006 |
| R0 | 35 (72.9%) | 63 (80.8%) | 23 (53.5%) |  |
| R1 | 13 (27.1%) | 15 (19.2%) | 20 (46.5%) |  |
| R2 | 0 (0.0%) | 0 (0.0%) | 0 (0.0%) |  |

Percentage (%) indicates the proportion of cases with a specific characteristic in all cases or strata of the amount of stromal collagen.

^a^The pathological stage was diagnosed based on Union for International Cancer Control (UICC) TNM classification, 8th Edition.

BMI, body mass index; CA19-9, carbohydrate antigen 19-9; CEA, carcinoembryonic antigen; GEM, gemcitabine; PDAC, pancreatic ductal adenocarcinoma.

**Supplemental Table S5.** Patient characteristics in relation to the amount of stromal collagen in PDAC in the S-1 group.

| **Characteristics** | **All patients** | **Tumor-stromal collagen** | | ***P* value** |
| --- | --- | --- | --- | --- |
|  | (N = 78) | Low (N = 40) | High (N = 38) |  |
| Age (years), median (range) | 69 (40-82) | 70 (40-82) | 69 (50-80) | 0.55 |
| Sex |  |  |  | 0.35 |
| Male | 48 (61.5%) | 27 (67.5%) | 21 (55.3%) |  |
| Female | 30 (38.5%) | 13 (32.5%) | 17 (44.7%) |  |
| BMI (kg/m^2^), median (range) | 20.9 (14.3-33.2) | 20.9 (14.3-33.2) | 21.0 (17.2-32.3) | 0.71 |
| Family history of PDAC |  |  |  | 0.67 |
| Present | 5 (6.4%) | 2 (5.0%) | 3 (7.9%) |  |
| Absent | 73 (93.6%) | 38 (95.0%) | 35 (92.1%) |  |
| Alcohol consumption |  |  |  | 0.20 |
| <50 (g/day) | 73 (93.6%) | 39 (97.5%) | 34 (89.5%) |  |
| ≥50 (g/day) | 5 (6.4%) | 1 (2.5%) | 4 (10.5%) |  |
| Smoking history |  |  |  | 0.02 |
| Present | 45 (57.7%) | 18 (45.0%) | 27 (71.1%) |  |
| Absent | 33 (42.3%) | 22 (55.0%) | 11 (28.9%) |  |
| Diabetes mellitus |  |  |  | 0.82 |
| Present | 32 (41.0%) | 17 (42.5%) | 15 (39.5%) |  |
| Absent | 46 (59.0%) | 23 (57.5%) | 23 (60.5%) |  |
| CA19-9 |  |  |  | 0.19 |
| <37 (U/ml) | 19 (24.4%) | 7 (17.5%) | 12 (31.6%) |  |
| ≥37 (U/ml) | 59 (75.6%) | 33 (82.5%) | 26 (68.4%) |  |
| CEA |  |  |  | 0.46 |
| <5 (ng/ml) | 56 (71.8%) | 27 (67.5%) | 29 (76.3%) |  |
| ≥5 (ng/ml) | 22 (28.2%) | 13 (32.5%) | 9 (23.7%) |  |
| Pathological stage^a^ |  |  |  | 0.50 |
| Ia/Ib | 19 (24.4%) | 8 (20.0%) | 11 (28.9%) |  |
| IIa/Iib | 40 (51.3%) | 23 (57.5%) | 17 (44.7%) |  |
| III | 19 (24.4%) | 9 (22.5%) | 10 (26.3%) |  |
| T factor |  |  |  | 0.77 |
| T1 | 14 (17.9%) | 7 (17.5%) | 7 (18.4%) |  |
| T2 | 48 (61.5%) | 25 (62.5%) | 23 (60.5%) |  |
| T3 | 15 (19.2%) | 7 (17.5%) | 8 (21.1%) |  |
| T4 | 1 (1.3%) | 1 (2.5%) | 0 (0.0%) |  |
| Tumor location |  |  |  | 0.07 |
| Head | 49 (62.8%) | 30 (75.0%) | 19 (50.0%) |  |
| Body | 22 (28.2%) | 8 (20.0%) | 14 (36.8%) |  |
| Tail | 7 (9.0%) | 2 (5.0%) | 5 (13.2%) |  |
| Histological grade |  |  |  | 0.003 |
| Well/moderately differentiated | 66 (84.6%) | 29 (72.5%) | 37 (97.4%) |  |
| Poorly differentiated | 12 (15.4%) | 11 (27.5%) | 1 (2.6%) |  |
| Residual tumor status |  |  |  | 0.57 |
| R0 | 63 (80.8%) | 31 (77.5%) | 32 (84.2%) |  |
| R1 | 15 (19.2%) | 9 (22.5%) | 6 (15.8%) |  |
| R2 | 0 (0.0%) | 0 (0.0%) | 0 (0.0%) |  |
| Neoadjuvant chemotherapy |  |  |  | 0.74 |
| Present | 10 (12.8%) | 6 (15.0%) | 4 (10.5%) |  |
| Absent | 68 (87.2%) | 34 (85.0%) | 34 (89.5%) |  |
| *KRAS* mutation |  |  |  | 0.61 |
| Present | 75 (96.2%) | 39 (97.5%) | 36 (94.7%) |  |
| Absent | 3 (3.8%) | 1 (2.5%) | 2 (5.3%) |  |
| *TP53* alteration |  |  |  | 0.63 |
| Present | 53 (67.9%) | 26 (65.0%) | 27 (71.1%) |  |
| Absent | 25 (32.1%) | 14 (35.0%) | 11 (28.9%) |  |
| *CDKN2A*/p16 alteration |  |  |  | 0.07 |
| Present | 57 (73.1%) | 33 (82.5%) | 24 (63.2%) |  |
| Absent | 21 (26.9%) | 7 (17.5%) | 14 (36.8%) |  |
| *SMAD4* alteration |  |  |  | 0.82 |
| Present | 36 (46.2%) | 19 (47.5%) | 17 (44.7%) |  |
| Absent | 42 (53.8%) | 21 (52.5%) | 21 (55.3%) |  |

Percentage (%) indicates the proportion of cases with a specific characteristic in all cases or strata of the amount of stromal collagen.

^a^The pathological stage was diagnosed based on Union for International Cancer Control (UICC) TNM classification, 8th Edition.

BMI, body mass index; CA19-9, carbohydrate antigen 19-9; CEA, carcinoembryonic antigen; GEM, gemcitabine; PDAC, pancreatic ductal adenocarcinoma.

**Supplemental Table S6.** Patient characteristics in relation to the amount of stromal collagen in PDAC in the GEM group.

| **Characteristics** | **All patients** | **Tumor-stromal collagen** | | ***P* value** |
| --- | --- | --- | --- | --- |
|  | (N=43) | Low (N=24) | High (N=19) |  |
| Age (years), Median (range) | 70 (45-85) | 69.5 (48-79) | 70 (45-85) | 0.89 |
| Sex |  |  |  | 0.99 |
| Male | 22 (51.2%) | 12 (50.0%) | 10 (52.6%) |  |
| Female | 21 (48.8%) | 12 (50.0%) | 9 (47.4%) |  |
| BMI (kg/m^2^), Median (range) | 20.8 (15.0-31.2) | 21.0 (16.5-31.2) | 20.7 (15.0-26.0) | 0.91 |
| Family history of PDAC |  |  |  | 0.68 |
| Present | 6 (14.0%) | 4 (16.7%) | 2 (10.5%) |  |
| Absent | 37 (86.0%) | 20 (83.3%) | 17 (89.5%) |  |
| Alcohol consumption |  |  |  | 0.36 |
| <50 (g/day) | 39 (90.7%) | 23 (95.8%) | 16 (84.2%) |  |
| ≥50 (g/day) | 4 (9.3%) | 1 (4.2%) | 3 (15.8%) |  |
| Smoking history |  |  |  | 0.31 |
| Present | 12 (27.9%) | 5 (20.8%) | 7 (36.8%) |  |
| Absent | 31 (72.1%) | 19 (79.2%) | 12 (63.2%) |  |
| Diabetes mellitus |  |  |  | 0.51 |
| Present | 13 (30.2%) | 6 (25.0%) | 7 (36.8%) |  |
| Absent | 30 (69.8%) | 18 (75.0%) | 12 (63.2%) |  |
| CA19-9 |  |  |  | 0.99 |
| <37 (U/ml) | 13 (30.2%) | 7 (29.2%) | 6 (31.6%) |  |
| ≥37 (U/ml) | 30 (69.8%) | 17 (70.8%) | 13 (68.4%) |  |
| CEA |  |  |  | 0.71 |
| <5 (ng/ml) | 34 (79.1%) | 18 (75.0%) | 16 (84.2%) |  |
| ≥5 (ng/ml) | 9 (20.9%) | 6 (25.0%) | 3 (15.8%) |  |
| Pathological Stage^a^ |  |  |  | 0.13 |
| Ia/Ib | 8 (18.6%) | 6 (25.0%) | 2 (10.5%) |  |
| IIa/IIb | 25 (58.1%) | 15 (62.5%) | 10 (52.6%) |  |
| III | 10 (23.3%) | 3 (12.5%) | 7 (36.8%) |  |
| T factor |  |  |  | 0.09 |
| T1 | 5 (11.6%) | 4 (16.7%) | 1 (5.3%) |  |
| T2 | 28 (65.1%) | 16 (66.6%) | 12 (63.2%) |  |
| T3 | 6 (14.0%) | 4 (16.7%) | 2 (10.5%) |  |
| T4 | 4 (9.3%) | 0 (0.0%) | 4 (21.2%) |  |
| Tumor location |  |  |  | 0.42 |
| Head | 33 (76.7%) | 18 (75.0%) | 15 (78.9%) |  |
| Body | 5 (11.5%) | 2 (8.3%) | 3 (15.8%) |  |
| Tail | 5 (11.5%) | 4 (16.7%) | 1 (5.3%) |  |
| Histological grade |  |  |  | 0.99 |
| Well/moderately differentiated | 37 (86.0%) | 21 (87.5%) | 16 (84.2%) |  |
| Poorly differentiated | 6 (14.0%) | 3 (12.5%) | 3 (15.8%) |  |
| Residual tumor status |  |  |  | 0.76 |
| R0 | 23 (53.5%) | 12 (50.0%) | 11 (57.9%) |  |
| R1 | 20 (46.5%) | 12 (50.0%) | 8 (42.1%) |  |
| R2 | 0 (0.0%) | 0 (0.0%) | 0 (0.0%) |  |
| Neoadjuvant chemotherapy |  |  |  | 0.58 |
| Present | 3 (7.0%) | 1 (4.2%) | 2 (10.5%) |  |
| Absent | 40 (93.0%) | 23 (95.8%) | 17 (89.5%) |  |
| *KRAS* mutation |  |  |  | 0.36 |
| Present | 38 (88.4%) | 20 (83.3%) | 18 (94.7%) |  |
| Absent | 5 (11.6%) | 4 (16.7%) | 1 (5.3%) |  |
| *TP53* alteration |  |  |  | 0.99 |
| Present | 28 (65.1%) | 16 (66.7%) | 12 (63.2%) |  |
| Absent | 15 (34.9%) | 8 (33.3%) | 7 (36.8%) |  |
| *CDKN2A*/p16 alteration |  |  |  | 0.34 |
| Present | 27 (62.8%) | 17 (70.8%) | 10 (52.6%) |  |
| Absent | 16 (37.2%) | 7 (29.2%) | 9 (47.4%) |  |
| *SMAD4* alteration |  |  |  | 0.06 |
| Present | 16 (37.2%) | 12 (50.0%) | 4 (21.1%) |  |
| Absent | 27 (62.8%) | 12 (50.0%) | 15 (78.9%) |  |

Percentage (%) indicates the proportion of cases with a specific characteristic in all cases or strata of the amount of stromal collagen.

^a^The pathological stage was diagnosed based on Union for International Cancer Control (UICC) TNM classification, 8th Edition.

BMI, body mass index; CA19-9, carbohydrate antigen 19-9; CEA, carcinoembryonic antigen; GEM, gemcitabine; PDAC, pancreatic ductal adenocarcinoma.

**Supplemental Table S7.** Patient characteristics in relation to the amount of stromal collagen in PDAC in the untreated group.

| **Characteristics** | **All patients** | **Tumor-stromal collagen** | | ***P* value** |
| --- | --- | --- | --- | --- |
|  | (N = 48) | Low (N = 21) | High (N = 27) |  |
| Age (years), median (range) | 70 (50-84) | 69 (51-83) | 70 (50-84) | 0.91 |
| Sex |  |  |  | 0.99 |
| Male | 25 (52.1%) | 12 (57.1%) | 13 (48.1%) |  |
| Female | 23 (47.9%) | 9 (42.9%) | 14 (51.9%) |  |
| BMI (kg/m^2^), median (range) | 20.9 (16.1-27.6) | 20.9 (16.1-27.6) | 20.9 (17.1-27.3) | 0.55 |
| Family history of PDAC |  |  |  | 0.98 |
| Present | 3 (6.2%) | 1 (4.8%) | 2 (7.4%) |  |
| Absent | 45 (93.8%) | 20 (95.2%) | 25 (92.6%) |  |
| Alcohol consumption |  |  |  | 0.71 |
| <50 (g/day) | 39 (81.2%) | 18 (85.7%) | 21 (77.8%) |  |
| ≥50 (g/day) | 9 (18.8%) | 3 (14.3%) | 6 (22.2%) |  |
| Smoking history |  |  |  | 0.56 |
| Present | 24 (50.0%) | 9 (42.9%) | 15 (55.6%) |  |
| Absent | 24 (50.0%) | 12 (57.1%) | 12 (44.4%) |  |
| Diabetes mellitus |  |  |  | 0.14 |
| Present | 20 (41.7%) | 6 (28.6%) | 14 (51.9%) |  |
| Absent | 28 (58.3%) | 15 (71.4%) | 13 (48.1%) |  |
| CA19-9 |  |  |  | 0.99 |
| <37 (U/ml) | 12 (25.0%) | 5 (23.8%) | 7 (25.9%) |  |
| ≥37 (U/ml) | 36 (75.0%) | 16 (76.2%) | 20 (74.1%) |  |
| CEA |  |  |  | 0.99 |
| <5 (ng/ml) | 31 (64.6%) | 14 (66.7%) | 17 (63.0%) |  |
| ≥5 (ng/ml) | 17 (35.4%) | 7 (33.3%) | 10 (37.0%) |  |
| Pathological stage^a^ |  |  |  | 0.62 |
| Ia/Ib | 8 (16.7%) | 4 (19.1%) | 4 (14.8%) |  |
| IIa/IIb | 31 (64.6%) | 12 (57.1%) | 19 (70.4%) |  |
| III | 9 (18.8%) | 5 (23.8%) | 4 (14.8%) |  |
| T factor |  |  |  | 0.60 |
| T1 | 6 (12.5%) | 2 (9.5%) | 4 (14.8%) |  |
| T2 | 27 (56.3%) | 11 (52.4%) | 16 (59.3%) |  |
| T3 | 14 (29.2%) | 7 (33.3%) | 7 (25.9%) |  |
| T4 | 1 (2.1%) | 1 (4.8%) | 0 (0.0%) |  |
| Tumor location |  |  |  | 0.17 |
| Head | 38 (79.2%) | 19 (90.5%) | 19 (70.4%) |  |
| Body | 7 (14.6%) | 2 (9.5%) | 5 (18.5%) |  |
| Tail | 3 (6.3%) | 0 (0.0%) | 3 (11.1%) |  |
| Histological grade |  |  |  | 0.31 |
| Well/moderately differentiated | 44 (91.7%) | 18 (85.7%) | 26 (96.3%) |  |
| Poorly differentiated | 4 (8.3%) | 3 (14.3%) | 1 (3.7%) |  |
| Residual tumor status |  |  |  | 0.99 |
| R0 | 35 (72.9%) | 15 (71.4%) | 20 (74.1%) |  |
| R1 | 13 (27.1%) | 6 (28.6%) | 7 (25.9%) |  |
| R2 | 0 (0.0%) | 0 (0.0%) | 0 (0.0%) |  |
| Neoadjuvant chemotherapy |  |  |  | 0.68 |
| Present | 7 (14.6%) | 4 (19.0%) | 3 (11.1%) |  |
| Absent | 41 (85.4%) | 17 (81.0%) | 24 (88.9%) |  |
| *KRAS* mutation |  |  |  | 0.99 |
| Present | 44 (91.7%) | 19 (90.5%) | 25 (92.6%) |  |
| Absent | 4 (8.3%) | 2 (9.5%) | 2 (7.4%) |  |
| *TP53* alteration |  |  |  | 0.34 |
| Present | 34 (70.8%) | 13 (61.9%) | 21 (77.8%) |  |
| Absent | 14 (29.2%) | 8 (38.1%) | 6 (22.2%) |  |
| *CDKN2A*/p16 alteration |  |  |  | 0.57 |
| Present | 25 (52.1%) | 12 (57.1%) | 13 (48.1%) |  |
| Absent | 23 (47.9%) | 9 (42.9%) | 14 (51.9%) |  |
| *SMAD4* alteration |  |  |  | 0.24 |
| Present | 16 (33.3%) | 9 (42.9%) | 7 (25.9%) |  |
| Absent | 32 (66.7%) | 12 (57.1%) | 20 (74.1%) |  |

Percentage (%) indicates the proportion of cases with a specific characteristic in all cases or strata of the amount of stromal collagen.

^a^The pathological stage was diagnosed based on Union for International Cancer Control (UICC) TNM classification, 8th Edition.

BMI, body mass index; CA19-9, carbohydrate antigen 19-9; CEA, carcinoembryonic antigen; GEM, gemcitabine; PDAC, pancreatic ductal adenocarcinoma.
